# Cerebellar transcranial direct current stimulation reconfigurates static and dynamic functional connectivity of the resting-state networks

**DOI:** 10.1186/s40673-021-00132-6

**Published:** 2021-02-24

**Authors:** F. Grami, G. de Marco, F. Bodranghien, M. Manto, C. Habas

**Affiliations:** 1 Laboratoire LINP2 « Laboratoire Interdisciplinaire de Neurosciences, Physiologie et Psychologie : Activité physique, Santé et Apprentissages», UPL, Université Paris Nanterre, Nanterre, France; 2 Unité d’Etude du Mouvement GRIM, FNRS, ULB-Erasme, Route de Lennik, Bruxelles, Belgium; 3 Services de Neurosciences, UMons, 7000 Mons, Belgium; 4 Unité des Ataxies Cérébelleuses, Service de Neurologie, CHU-Charleroi, 6000 Charleroi, Belgium; 5 Service de Neuroimagerie, Centre Hospitalier National d’Ophtalmologie des 15-20, Quinze-Vingt, 28, rue de Charenton, 75012 Paris, France

**Keywords:** Transcranial direct stimulation, Cerebellum, Resting-state, Functional connectivity, Dynamics, Limbic network, Salience network, Default-mode network

## Abstract

**Background:**

Transcranial direct current stimulation (tDCS) of the cerebellum dynamically modulates cerebello-thalamo-cortical excitability in a polarity-specific manner during motor, visuo- motor and cognitive tasks. It remains to be established whether tDCS of the cerebellum impact also on resting-state intrinsically connected networks (ICNs). Such impact would open novel research and therapeutical doors for the neuromodulation of ICNs in human.

**Method:**

We combined tDCS applied over the right cerebellum and fMRI to investigate tDCS- induced resting-state intrinsic functional reconfiguration, using a randomized, sham-controlled design. fMRI data were recorded both before and after real anodal stimulation (2 mA, 20 min) or sham tDCS in 12 right-handed healthy volunteers. We resorted to a region-of-interest static correlational analysis and to a sliding window analysis to assess temporal variations in resting state FC between the cerebellar lobule VII and nodes of the main ICNs.

**Results:**

After real tDCS and compared with sham tDCS, functional changes were observed between the cerebellum and ICNs. Static FC showed enhanced or decreased correlation between cerebellum and brain areas belonging to visual, default-mode (DMN), sensorimotor and salience networks (SN) (p-corrected < 0.05). The temporal variability (TV) of BOLD signal was significantly modified after tDCS displaying in particular a lesser TV between the whole lobule VII and DMN and central executive network and a greater TV between crus 2 and SN. Static and dynamic FC was also modified between cerebellar lobuli.

**Conclusion:**

These results demonstrate short- and long-range static and majorly dynamic effects of tDCS stimulation of the cerebellum affecting distinct resting-state ICNs, as well as intracerebellar functional connectivity, so that tDCS of the cerebellum appears as a non-invasive tool reconfigurating the dynamics of ICNs.

## Background

Amongst non-invasive brain stimulation techniques, transcranial direct current stimulation (tDCS) has gained increasing popularity in neurosciences and medical research. In particular, this method has been recently used to investigate the functional role of the sensorimotor and cognitive cerebellar networks [1]. tDCS exerts a direct, local but only partially known effect upon the cortical stimulation site, and an indirect distal effect linked to this site-recipient afferent pathways. Within the stimulated cortical volume, tDCS modulates the resting membrane potential of glutamatergic neurons during the stimulation phase, and causes afterwards long-lasting synaptic after-effects due to interneuronal and neuromodulatory influences, in a polarity-specific manner [2]. For instance, anodal tDCS increases neuronal excitability and synaptic strength (effect on neuronal plasticity), whereas cathodal tDCS provokes an opposite effect [3]. Regarding tDCS of the cerebellum, anodal (vs cathodal) stimulation applied over the cerebellar cortex would preferentially enhance (vs reduce) cerebellar brain inhibition (CBI), and modulates sensorimotor and cognitive functions overall [3]. Cerebellar tDCS affects not only overt motor/cognitive abilities such as motor execution and adaptation, working memory, procedural learning and linguistic processing and emotion recognition, but influences also the brain resting-state [1, 3–5].

Intrinsically-connected, cerebello-cortical closed loops encompass the sensorimotor, language, executive, dorsal attentional/saccadic control, limbic salience and default-mode networks [6, 7]. Although non invasive cerebellar stimulation (including transcranial magnetic stimulation (TMS) of the cerebellum) changes functional connectivity (FC) within default-mode network (DMN) and dorsal attentional network (DAN), little is known about static and especially dynamic resting state reconfiguration caused by cerebellar tDCS. First, tDCS might simultaneously alter functional connectivity of several cerebello-cortical circuits because since the main cerebellar target of tDCS is the lobule VII, which is involved in several circuits [6] and, to a lesser extent, the more rostral and remote from the electrode lobules VI and VIII [3]. Second, most of the fMRI studies have focused on the static pattern of intrinsic connectivity, resorting, in particular, to data-driven, seed-based correlational or Independent Component Analyses (ICA) [8]. However, these methods assume the stationarity of the FC throughout the entire scan period, although the brain resting-state presents spontaneous, time-varying within- and between-network associations [9]. These non- stationary changes of the brain resting-state can be captured by complementary methods such as sliding window based FC, with temporal limitations due to TR and hemodynamic response durations and to statistical constraints. Notwithstanding, dynamical FC provides a better and more accurate description of the spatio-temporal-varying pattern of brain intrinsic connectivity.

We aimed to explore the static and dynamic brain functional after-effects of tDCS applied over the right cerebellar hemisphere in healthy subjects, during the brain resting-state. We specifically focused on the FC of the right lobule VII (crus 1 and 2, VIIb) whose anatomical localization within the posterior cranial fossa allows a direct stimulation. Interestingly, lobule VII is massively involved in the following brain networks in central executive network (CEN; also called right and left fronto-parietal networks), DMN, and salience network (SN) [6].

## Materials and methods

### Subjects

Twelve healthy right-handed volunteers (mean age ± SD: 28 ± 2 years; 9 males and 3 females) participated in this study. None of them had history of neurological, psychiatric or vascular disease, and of alcohol or drug abuse. All of them gave their informed consent. The protocol was approved by the Ethics Committee (Study number: 2014-A01580–47). All participants received 50 euros compensation for their participation in the current study.

### Transcranial direct current stimulation

Outside of the scanner, real tDCS stimulation and Sham stimulation were randomly delivered to each volunteer. Each stimulation session was immediately followed by a MRI scanning. Saline- soaked, rectangular-shape electrode sponges were used for both stimulation conditions. The anodal electrode (size: 5 × 6 cm) and the reference cathodal electrode (size: 9 × 7 cm) were placed over the right posterior cerebellar hemisphere, i.e. halfway between subject’s mastoid and inion, and over the left acromion, respectively [10]. During the real tDCS stimulation, each participants received 1,5 mA current for a total of 20 min including an initial 30 s ramp-up [3]. Sham session consisted of a 30 s ramp-up from 0 mA to 1.5 m followed by a 30 s ramp-down to 0 mA; no current was delivered during the next 19 min. Impedance of the electrodes was kept below 10 kΩ.

### Imaging protocol

Immediately after each randomized condition, the volunteers underwent a functional MRI scan to record their resting-state brain activity. Each volunteer laid in supine position with eyes-closed. During the first MRI scan, T1-weighted anatomical images were also acquired.

### MRI data acquisition

Structural and functional data were obtained on a whole-body 3 T scanner (Siemens, Skyra, Erlangen, Germany) with a 64-channel head coil. T1-weighted gradient-echo images were acquired with the following parameters: TE, 2.5 ms; TR, 2200 ms; TI, 900 ms, flip angle, 8°; voxel.

size, 0.9× 0.9 mm^2^ (FOV, 230 mm^2^), slice thickness, 0.9 mm, 176 axial slices; GRAPPA acceleration factor, 2. Sixty contiguous axial slices, multi-band (SMS) T2*-weighted gradient echo- planar images (echo time 30 ms, repetition time 1000 ms, flip angle 90°, spatial resolution 2.5 × 2.5. × 2.5 mm, acceleration factor 2), were acquired to encompass the whole brain. One hundred eighty four volumes were acquired with four “dummy” volumes recorded at the start of the one-run session to allow for steady-state magnetization.

## Methods

### Preprocessing of the fMRI data

The preprocessing was performed using the SPM 12 software (http://www.fil.ion.ucl.ac.uk/spm) implemented in Matlab. In each data set, for T1 equilibrium, the first 10 volumes were discarded. All EPI images were: corrected for slice timing and motion correction, temporally and spatially smoothed with 5 mm full width half maximum Gaussian kernel and co-registered with the T1 anatomical images and then spatially normalized using a template brain of Montreal Neurological Institute (MNI).

### Static functional connectivity (sFC) analysis

The FC analysis was carried out using CONN toolbox (v.19c). For each participant, principal components were extracted from white matter and cerebrospinal fluid time-series in order to reduce noise based on the anatomical CompCor approach [11]. These components and head movements’ parameters were added as confounds in the denoising step. We conducted seed-to-voxel analyses using 3 a priori region-of-interest (ROI) corresponding to the stimulated right cerebellar lobule VII including crus 1, crus 2 and lobule 7b. The cerebellar ROIs were obtained from the AAL atlas [12]. At the first level analysis, resting-state BOLD signal time-series were extracted from each seed region and correlated with every voxel in the brain. Each participant’s maps were brought into a second level analysis to examine the effects of anodal tDCS > Sham tDCS. The resulting sFC were reported significant at a cluster-level-threshold of *p* < 0.05 false discovery rate (FDR), corrected and a voxel count ≥100.

### Dynamic functional connectivity (dFC) analyses

A sliding window ROI-to-ROI correlation analysis was applied to seek for temporal variations in FC between sublobuli of the right cerebellar lobule VII and: 1. specific nodes of ICNs, during the resting-state, and 2. other cerebellar lobules.

Time-courses were segmented into 36-s windows, sliding the onset of each window by 18 s for a total of 10 windows, in accordance with previous dynamic studies [13, 14]. The duration of sliding windows was selected to optimize the balance between capturing rapidly shifting dynamic relationships (with shorter windows) and achieving reliable estimates of the correlated activity between regions (with longer windows). Then, a band-pass filtering (0.0278–0.10 Hz) was applied to remove high-frequency activity related to cardiac and respiratory activity, and low-frequency activity with a period that exceeds the duration of sliding windows used in dynamic analyses.

Dynamic temporal variability, between two ROIs, were calculated using a regression model using the generalized psychophysiological interaction approach (gPPI), which allows to explore brain areas which increase/decrease their couplings with the seed region of the cerebellar lobule VII, massively structurally and functionally interconnected with associative cortices. The regions of Salience network (SN), central executive network (CEN), visual network (VN), sensorimotor network (SMN) and dorsal attention network (DAN) were examined. The degree of temporal variability in FC is defined as the standard deviation (SD) in bivariate regression measures between two ROIs. To test the hypothesis that brain activity after tDCS stimulation (versus sham) differed on dynamic resting state connections, a paired t-test was performed. Results were reported significant at a threshold of *p* < 0.05, FDR corrected.

The underlying patterns of dFC across sliding windows that contributed to significant tDCS after- effects, were quantified using descriptive statistics based upon the frequency of positive or negative z scores. The z score is defined as:

$$ Z=\frac{X_{tDCS}-{M}_{sham}}{S_{sham}} $$

where *XtDCS* denotes the tDCS-related β value within each time window in real tDCS condition, *Msham* represents the mean of β values across all windows in the sham condition, and *Ssham* is the standard deviation of β values of all windows in the sham condition. The β values correspond to the regressors of the linear equation expressing the brain signal within the region-of-interest with respect of the mean signal and the differential effect induced by real tDCS and sham stimulations.

More specifically, for each participant, the proportion of windows was computed by transforming the connectivity values to z-scores and sorted into five intervals of increasing strength: high negative (z < − 0.5), moderate negative (− 0.5 ≤ z < − 0.25), low negative / positive (− 0.25 ≤ z < 0.25), moderate positive (0.25 < z ≤ 0.5) and high positive (z > 0.5). Finally, the average proportion of windows in which FC fell within each range was calculated. Data normality in z-score was tested using Shapiro-Wilk test.

## Results

### Static analysis

**Fig. 1:**
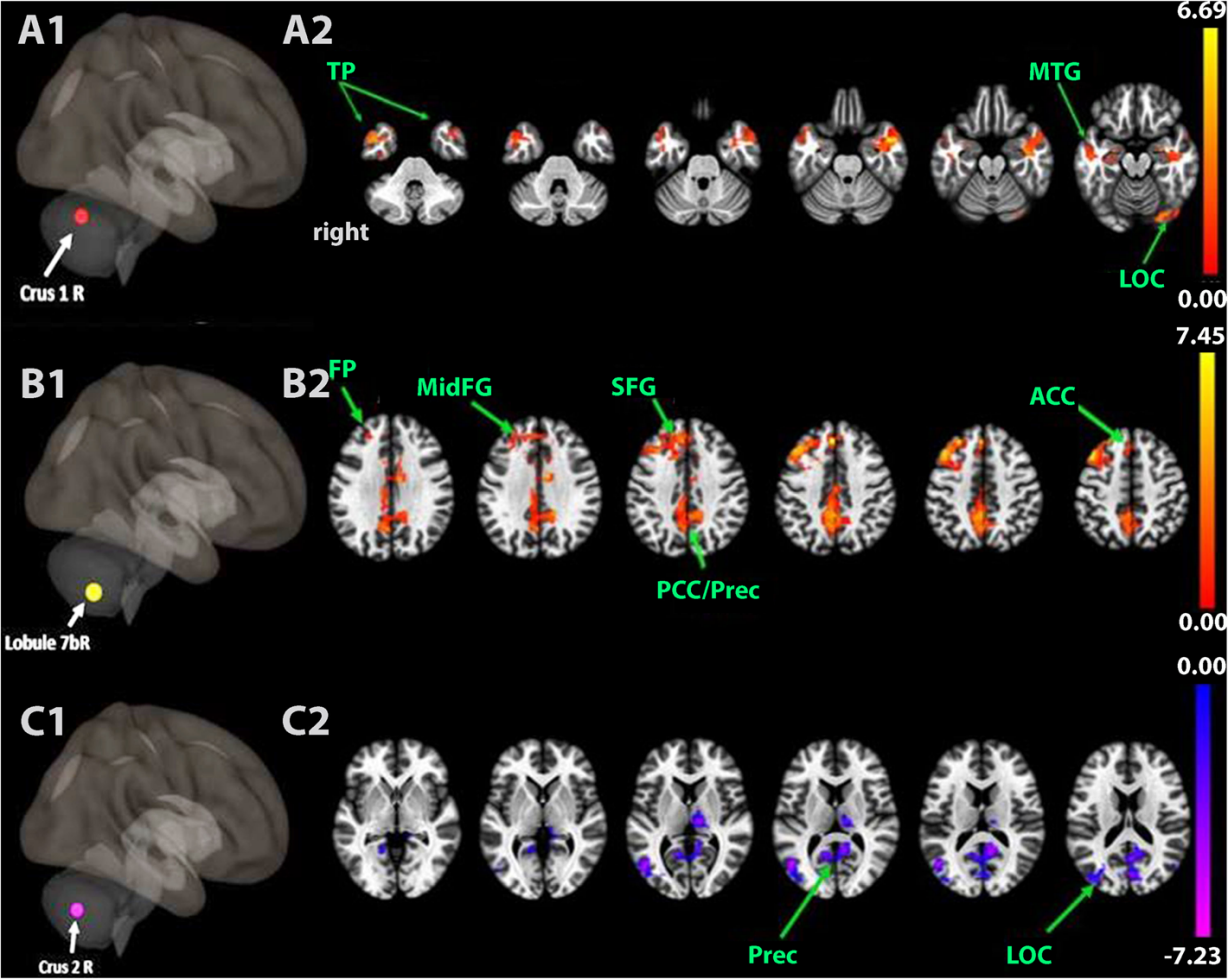
Static resting-state functional connectivity map between seeded right lobule VII and nodes of intrinsic networks (*p*-FDR < 0.05, cluster-level) after real tDCS stimulation compared with sham tDCS. A1-C1. Schematic lateral 3D-view showing the seeded cerebellar lobules. A2-C2. Axial slices passing through the brain. A2. Increased functional connectivity between crus 1 and visual/temporal regions. B2. Increased functional connectivity between lobule 7b and nodes belonging to SN (ACC), DMN (PCC, Precuneus) and CEN (FP, MidFG). C2. decreased functional connectivity between crus 2 and visual/ DMN regions. The colored bars represent the z-value

Table 1 anf Fig. 1 display significant (p-FDR < 0.05, cluster-level) of static FC between seeded sublobuli of lobule VII and nodes of ICNs, after real tDCS stimulation and compared with sham tDCS. First, increased FC (positive correlation) was observed between crus 1 and: ipsilateral VN/DMN (posterior cingulate, temporal and hippocampal cortices), and between lobule 7b and nodes of SN (ACC, paracingulate cortex), DMN (precuneus) and CEN (prefrontal cortex). Second, decreased FC (negative correlation) was found between crus 1 and contralateral VN/ipsilateral SMN/DMN (precuneus), and between crus 2 and contralateral VN/DMN.

**Table 1:** Right cerebellum: real tDCS stimulation > SHAM stimulation

ROI	Clusters (x,y,z)*	Size (k)	Brain Region	Voxel count Right Left		Size ***p***-FDR
**Positive correlation**						
**Crus 1**	- 46 4–26	3966				0.001
			OP	586	**–**	
			iLOC	464	**–**	
			TP	454	–	
			OFusG	428	–	
	−8 -38 34	2668				0.009
			TP	–	553	
			PPC	–	286	
			aMTG	–	115	
			Hippocampus	–	105	
**Lobule 7b**	−02 34 42	4675				0.0004
			FP	–	1118	
			MidFG	–	970	
			SFG	–	477	
			PaCiG	–	384	
			FP	238	–	
			ACC	174		
	−14 -56 24	4095				0.0006
			Precuneous	1550		
			PCC	978		
			ACC	188		
**Negative correlation**						
**Crus 1**	- 48 -68 10	6149				0.000
			sLOC	–	872	
			Precuneous	649		
			PreCG	512	**–**	
			sLOC	432	–	
			iLOC	–	410	
			Thalamus	364	–	
			Cuneal	227	–	
			LG	–	180	
			PostCG	131	–	
			Cuneal	–	109	
			ICC	–	100	
**Crus 2**	−26 -68 18	2609				0.02
			Precuneous	510		
			sLOC	–	502	
			iLOC	–	247	
			LG	–	201

Table 2 displays significant (p-FDR < 0.05, cluster-level) of static FC between seeded sublobuli of lobule VII and other cerebellar lobuli, after real tDCS stimulation and compared with sham tDCS. Increased FC was detected between crus 1–2 and the anterior lobe including the vermis, whereas bilateral decreased FC was noted preferentially between right lobule 7 and the neocerebellum (lobules 7–9) including the uvula.

**Table 2:** Right cerebellum: real tDCS stimulation > SHAM stimulation

ROI	Brain Region		Side	T value	***p***-FDR
	Vermis	Hemisphere			
**Positive correlations**					
Crus 1	Lobule 3	–	–	4.87	0.0002
	–	Lobule 4 5	R	1.98	0.03
	Lobule 6 (Declive)	–	–	2.34	0.01
Crus 2	Lobule 6	–	–	3.76	0.001
	–	Lobule 6	L	2.22	0.02
**Negative correlations**					
Crus 1	Lobule 7		–	−3.80	0.001
	–	Lobule7b	R	−1.93	0.03
	–	Lobule 8	L	−3.81	0.001
	–	Lobule 9	L	− 3	0.006
	Lobule 9 (Uvula)	–	–	−6.98	0.00001
	–	Lobule 10	R	−3.32	0.006
			L	−2.88	0.007
Crus 2		Crus 2	L	−1.87	0.04
		Lobule 8	L	−2.19	0.02
	Lobule 9 (Uvula)	–	–	− 2.16	0.02
Lobule 7b	–	Crus 1	R	−1.93	0.03
	–	Crus 2	L	−2.06	0.03
	–	Lobule 6	R	−2.92	0.006
	–	Lobule 8	L	−2.54	0.01
	Lobule 9 (Uvula)	–	–	−2.31	0.02
	–	Lobule 10	L	−2.34	0.01

### Dynamic analyses

#### Temporal variability (standard deviation across averaged sliding windows)

Tables 3 displays significant (p-FDR < 0.05, cluster-level) increased and decreased temporal variability of BOLD signal between cerebellar sublobuli of right and the rest of the brain, respectively, induced by anodal tDCS compared with sham tDCS. First, tDCS modulated FC between the whole lobule VII and all the main ICNs. Second, the one-sided lobule VII exerted a bilateral influence upon nodes of each ICN. Third, the temporal variability of cerebello-ICNs FC was preferentially decreased by tDCS real stimulation, with the notable exception of crus 2 and SMN and SN nodes (anterior insula and ACC), which displayed together increased temporal variability, whereas the reverse pattern of temporal variability was observed between the left lobule VII and the functionally connected ICNs. Fourth, weaker temporal variability was found between crus 1 and all the networks included in this study; increased temporal variability was mainly found between crus 2 and SN, and between crus 2 and CEN. Fifth, temporal variability was diminished between crus 1 and 2 and their contralateral cerebellar homologue.

**Table 3:** Right cerebellum: real tDCS stimulation > SHAM stimulation (*N* = 12)

Seed ROI	ROIs	Side	MNI-Coordinates x y z	T	Size ***p***-FDR
**Default Mode Network**					
Crus 1	PPC (BA 39)	L	−39 -77 33	−1.83	0.04
	PPC	R	47–67 29	− 1.98	0.03
Crus 2	PPC	L	−39 -77 33	−2.34	0.01
Lobule 7b	PPC	L	−39 -77 33	−3.98	0.001
	MPFC (BA 10)	–	1 55–3	−2.26	0.02
**Sensorimotor Network**					
Crus 1	lM1/S1	L	−55 -12 29	−2.40	0.01
Crus 2	lM1/S1	L	−55 -12 29	**+ 1.90**	0.04
	sM1/S1	–	0–31 67	**+ 2.18**	0.02
Lobule 7b	lM1/S1	L	−55 -12 29	**+ 2.58**	0.01
	sM1/S1	–	0–31 67	**+ 2.15**	0.02
**Visual Network**					
Crus 1	Occ (BA 17)	–	0–93 -4	**+ 1.82**	0.04
	Occ	–	2–79 12	−1.96	0.03
Crus 2	Occ	–	0–93 -4	**+ 2.79**	0.008
	lOcc (BA 19)	R	38–72 13	−2.42	0.02
	lOcc	L	−37 -79 10	−2.23	0.02
Lobule 7b	Occ	–	0–93 -4	− 3.11	0.004
	Occ	–	2–79 12	−3.20	0.004
**Frontoparietal Network or Central executive Network**					
Crus 1	PPC (BA 39)	R	52–52 45	−3.06	0.005
Crus 2	PPC	R	52–52 45	−2.11	0.02
	PPC	L	−46 -58 49	**+ 2.12**	0.02
Lobule 7b	PPC	R	52–52 45	−2.47	0.01
	PPC	L	−46 -58 49	−2.27	0.02
**Salience Network**					
Crus 1	SMG	R	62–35 32	−2.05	0.03
	SMG	L	−60 -39 31	−2.97	0.006
Crus 2	aInsula	R	47 14 0	**+ 1.99**	0.03
	aInsula	L	−44 13 1	**+ 2.3**	0.02
	SMG	R	62–35 32	**+ 1.92**	0.03
	SMG	L	−60 -39 31	**+ 1.98**	0.03
	ACC	–	0 22 35	**+ 1.85**	0.04
Lobule 7B	SMG	R	62–35 32	−3.01	0.005
	PFC	R	32 46 27	−2.93	0.006
	PFC	L	−32 45 27	−2.35	0.01
	ACC	–	0 22 35	−1.81	0.04
**Dorsal Attention Network**					
Crus 1	IPS (BA 7)	R	39–42 54	−2.08	0.03
	FEF	L	−27 -9 64	−2.26	0.02
Crus 2	FEF	R	30–6 64	**+ 2.01**	0.03
Lobule 7b	IPS	L	−39 -43 52	**+ 2.55**	0.01

Tables 4 displays significant (p-FDR < 0.05, cluster-level) increased and decreased temporal variability of BOLD signal between cerebellar sublobuli of right and other cerebellar lobuli, respectively, induced by anodal tDCS compared with sham tDCS. Decreased and increased dynamic FC is observed between lobule 7 and the rest of the cerebellum. In particular, decreased TV is noted between right crus 1–2 and their left homotopic regions, whereas increased TV was observed between right and left sublobule 7b.

**Table 4:** Right cerebellum: real tDCS stimulation > SHAM stimulation

Seed ROI	ROIs		Side	T-Value	Size ***p***-FDR
	Vermis	Hemisphere			
**Increased Temporal Variability**					
Crus 1	–	Lobule 4 5	R	3.79	0.001
	–	Lobule 4 5	L	2.1	0.02
	–	Lobule 9	R	3.22	0.004
Crus 2	Lobule 3	–	–	1.99	0.03
	–	Lobule 7b	L	1.96	0.03
	Lobule 8 (Pyramis)	–	–	2.25	0.02
	–	Lobule 10	L	2.07	0.03
Lobule 7b	–	Lobule 7b	L	2.21	0.02
	–	Lobule 8	R	2.54	0.01
	Lobule 8 (Pyramis)	–	–	2.75	0.009
	Lobule 9 (Uvula)	–	–	2.52	0.01
**Decreased Temporal Variability**					
Crus 1	–	Crus 1	L	−4.38	0.0005
	Lobule 7	–	–	−2.64	0.01
	Lobule 9 (Uvula)	–	–	−2.64	0.01
Crus 2	–	Crus 1	L	−3.41	0.002
	–	Crus 2	L	−2.87	0.007
	Lobule 4 5 (Culmen)	–	–	−2.78	0.007
Lobule 7b	–	Lobule 3	R	−1.98	0.03
	Lobule 4 5 (Culmen)	–	–	−3.21	0.004
	–	Lobule 9	L	−2.81	0.008

#### Frequency of windows (z-scores)

##### DMN nodes

**Fig. 2:**
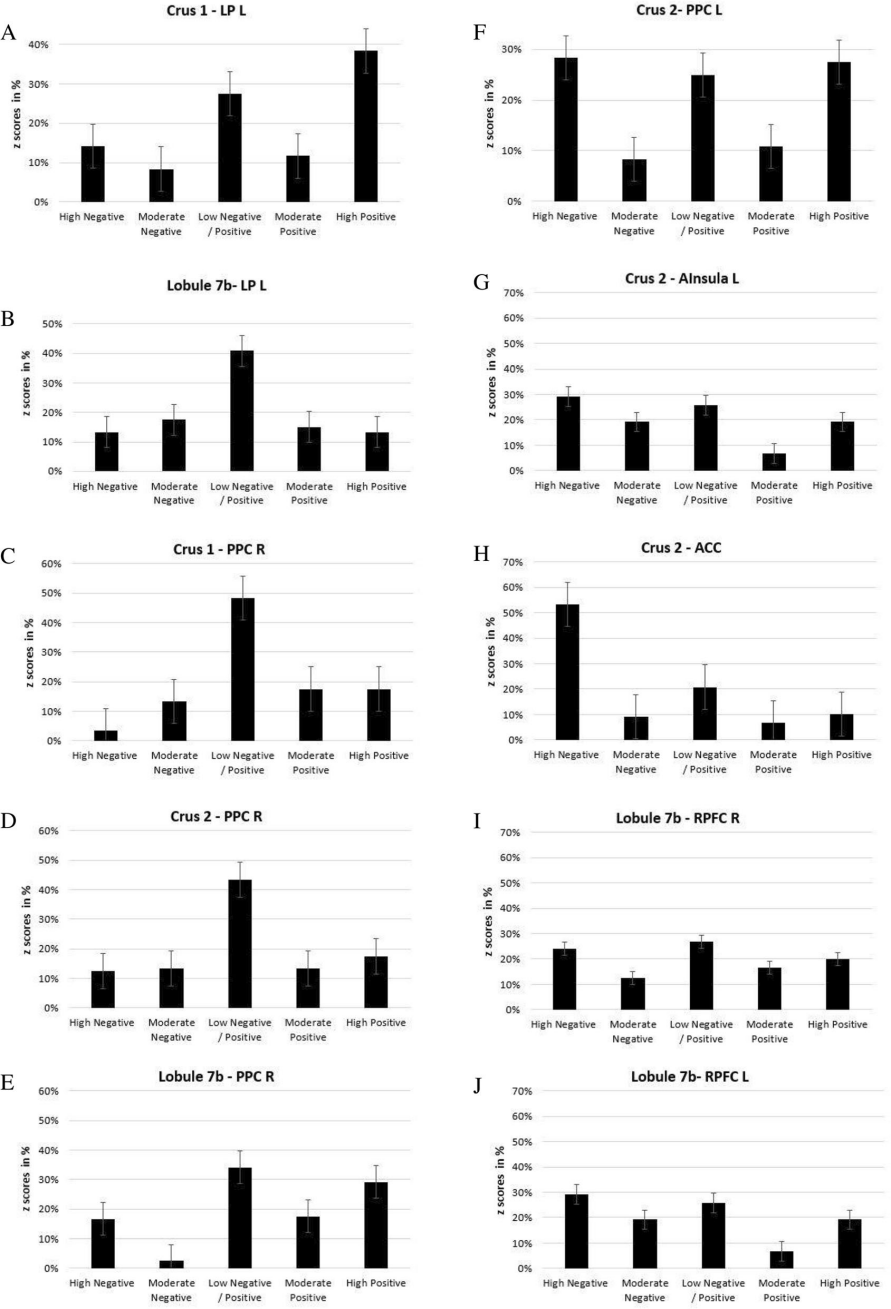
Histograms describing the proportion of windows in which the z-scores fell within particular ranges for the *N* = 12 subjects: high negative (z < −0.5), moderate negative (−0.5 < z < −0.25), low negative/positive (−0.25 < z < 0.25), moderate positive (0.25 < z < 0.5), and high positive (z > 0.5). **a**-**b**, z-score distribution between crus 2/7b and left LP. **c**-**d**-**e**, z-score distribution between crus 1–2/7b and right PPC. **f**, z-score distribution between crus 2 and left PPC. **g**, z-score distribution between crus 2 and left anterior insula. H, z-score distribution between crus 2 and right PFC; **i**, z-score distribution between right lobule 7B and right PFC **j**, z-score distribution between right lobule 7B and left PFC

The decreased average temporal variability observed between crus 2 and left LP (Table 2) was related to more frequent sliding-windows of moderate to high positive z scores (greater than 75%) and very few sliding-windows in which the z scores were highly negative (less than 5%) (Fig. 2a). A similar pattern of temporal variability was also found between crus I and left LP.

The decreased average temporal variability observed between lobule 7b and left LP (Table 2) showed a quasi-symmetric pattern around z = 0 between the negative and positive z-scores values (60% in total) (Fig. 2b).

##### CEN nodes

The histograms (Fig. 2c and d) showed a weak temporal variability between crus 1 and 2 and right PPC accompanied by increased frequency of windows in which the z-scores varied around low/moderate negative/positive values (more than 75%).

The decreased temporal variability observed between lobule 7b and right PPC (Table 2) was related to more frequent sliding-windows of moderate to high positive z scores (50%) and very few sliding- windows in which the z scores were highly negative (less than 15%) (Fig. 2e).

The increased temporal variability observed between crus 2 and CEN (posterior parietal cortex) was related to more frequent windows of high negative and positive z-score (56% of windows) to moderate negative/positive z-score (25% of windows) (Fig. 2f).

##### SN nodes

The temporal variability observed between crus 2 and SN nodes (left anterior insula and ACC) (Table 2) was related to more frequent windows of high negative z-score (60% of windows) (Fig. 2g and h). The temporal variability between lobule 7b and right RPFC (Fig. 2i) was related to similar proportions of sliding-windows in which the z-scores were moderate negative/positive and high negative/positive. A similar pattern of temporal variability was also observed between lobule 7b and left RPFC (Fig. 2j).

## Discussion

Our study shows that anodal stimulation of the right cerebellar cortex modulates static and, especially, dynamic resting-state FC between the stimulated right lobule VII and ICNs. In particular, we show for the first time that tDCS caused greater temporal variation of BOLD signal between crus 2 and SN/DAN/SMN and between lobule 7b and DAN/SMN whereas tDCS induced preferentially lesser temporal variability between crus 1 and ICNs and between crus 2 and DMN/CEN/VN. Moreover, our study also reveals that tDCS alters differentially the cerebello- cortical time variability with respect to low-to-high β values converted into z-score, whatever the time variability might be increased or decreased compared to sham. More precisely, increased time variability is accompanied by enhanced frequency of windows in high z-scores (60%), and dimished time variability is accompanied by increased frequency of windows in small and moderate z-cores. We also noticed tDCS-induced between-lobuli FC reorganization within the cerebellum.

tDCS stimulation applied over the right neocerebellum modulates all the tested ICNs. The cerebellar region stimulated by the anodal electrode mainly corresponds to the underlying hemisphere of lobule VIIab where the electric field strength is known to reach focally its maximal value [15], even if more discrete and anatomically restricted stimulation of adjoining lobules VI and VIII cannot be ruled out. Moreover, the lobule VII occupies 49, 75% of the whole cerebellar volume [16], and is massively structurally and functionally interconnected with associative cortices. More precisely, functional coherence was found between crus 1–2 and prefrontal/ parietal/ temporal/ posterior cingulate cortices (BA 8/9/10/46), between crus 1 and rostral inferior parietal cortex and ACC, between crus 2-lobule VIIb and posterior parietal cortex (BA 39 including the overlying intraparietal sulcus, precuneus) [17–19] and between lobule VIIb and DAN [4]). Cerebellum is also functionally linked to the insula [20]. Cerebello-cortical FC was bilateral with a contralateral predominance. At the network level, the lobule VII constitutes a hub being part of the CEN, DMN, SN [6] and VN [21]. It is noteworthy that the CEN, also called frontoparietal network (FPN), proved to be two-fold overrepresented within the cerebellar cortex [22].

We have found, with the static analysis, bilateral dynamic FC connections between lobule VII and DMN, CEN, SN, VN and DAN. Bilateral FC between cerebellum and cerebral cortex have been reported previously [6]. Such bilaterality may rely, despite the massive projection of the dentate nuclei to the contralateral thalamus through the superior cerebellar peduncle, on recrossed thalamic projection to ipsilateral thalamus [23], on ipsilateral collaterals arising from pontine or reticular nuclei, to transcallosal connection, or to another brain relay not accounted by our predefined regions-of-interest used in the connectivity analyses [8]. Furthermore, the parallel fibers may also participate in the changes observed, by linking cerebellar cortical sites. It is noteworthy that Park et al. [24] has demonstrated tDCS-induced enhancement of interhemispheric connectivity during the resting state.

CEN is subdivided into right and left frontoparietal networks likely subserving, besides general executive functions, more specialized functions in visuospatial or linguistic/logical domains, respectively. tDCS-induced temporal variability was increased between crus 2 and left CEN, whereas temporal variability was decreased between crus 2 and right CEN, pointing out a dual influence of crus 2 upon CEN. However, decreased temporal variability was observed between CEN and the other cerebellar sublobuli. On this vein, anodal tDCS stimulation over the right parietal cortex yielded to enhanced static FC between cerebellum (crus 1 and 2) and precuneus (DMN) and contralateral CEN [25].

We have found changes of static and dynamic FC between lobule VII and SMN/VN, although no anatomical nor direct known functional links exist between lobule VII and motor or visual areas. Two explanations can be proposed: either a “hidden” brain node or spread of electrical stimulation to nearby lobules VI and VIII for SMN. There might be a direct or indirect recruitment of the oculomotor vermis of lobules VI caudal and VII, and of lobules IV-V through intracerebellar functional connection [26]. Kelly and Strick [27] also traced in monkey connections between lobule VIIb and M1. In addition, a contribution of brainstem relays cannot be ruled out.

If the main effect of cerebellar tDCS consisted in temporally stabilizing FC between cerebellum and ICNs, we measured a greater temporal variability between crus 2 and the main nodes of SN (anterior insula, ACC and the supramarginal cortex). SN plays a major role in switching activity between DMN involved in task-negative mind wandering and CEN. Resting state high activity of SN can also be associated with greater FC between DMN and CEN [28].

Anodal tDCS can transiently alter alpha, beta and gamma brain oscillations [29–32]. In rat, crus 1 processed phases and phase differences between prefrontal and hippocampal oscillations [33] suggesting a cerebellar role in timing and temporal interaeal coordination. Therefore, tDCS may modulate within- and cross-network synchronization which may concern predominantly DMN and CEN. During the resting state, using co-activation pattern analysis, Karahanoglu and van De Ville [34] showed that: (1) DMN including crus 1 had the longest dwell time, (2) DMN and SN were anticorrelated, and (3) DMN and CEN activations tended to co-occur with the same or opposite (posterior DMN) sign. Moreover, DMN, especially the posterior parietal cortex, transiently correlated in the beta band with other networks [35]. Therefore, it can be hypothesized that anodal tDCS might modulate brain oscillations involved in dynamic synchronization/desynchronization of DMN, CEN and SN, which contributed to reinforce cerebellum-DMN/CEN FC and to diminish crus 2-SN/SMN/DAN FC. In particular, the more flexible interaction between crus 2 and SN, and conversely, the reverse pattern between crus1 and SN, could reflect a dual and antagonistic cerebellar control of lobule VIIa upon the SN ability to switch between CEN and DMN activity, likely in relation to different prefronto-cerebellar afferents. Of interest, crus 2 showed predominantly increased or dual increased/decreased dynamic connectivity with ICNs (except with DMN). Crus 2 should participate in dynamic switching between ICNs or between specific nodes of ICNs all the more easily that it is functionally connected with prefrontal, parietal and cingulate cortices, and with the DMN/SN/CEN.

Dynamic FC exhibited more widespread tDCS-induced effects of lobule VII onto ICNs than static FC. We have found agreement between static and dynamic results for crus1 and lobule VIIb which manifested preferentially increased FC. However, after tDCS, crus 2 showed decreased static FC with DMN whereas dynamic FC was weakened. Further studies are required to reconcile these discrepant results. Moreover, dynamic FC between lobule VIIb and ICNs had the highest FDR p- value (*p* ≤ 0.01). Lobule VIIb is involved, at least, in executive, linguistic and visual working memory [6, 7], and is in functional coherence with CEN [6], SN [6] and DAN [7]. Lobule VIIb thus seemed to constitute an important cerebellar hub controlling ICNs.

tDCS also changed static and dynamic FC between cerebellar lobuli. Such changes might modulate cooperation between ICNs and between homotopic lobuli within the cerebellum.

Our results should be replicated and complemented with a larger population, a longer duration of fMRI recording, a characterization of the thought content during the mind wandering, and neuropsychological tests performed before and after tDCS/MRI sessions to seek for tDCS- induced transient modifications, for example, of executive functions. Although a larger population is required to address generalization of our results to the general population, our data clearly showed that tDCS caused important transient FC reorganization. Finally, the variable z-score distributions between lobule 7 and ICNs observed during tDCS versus sham requires also further studies to grasp its functional and underlying neurophysiological meaning.

In conclusion, anodal tDCS over the right cerebellum causes static and mostly dynamic changes in resting state FC characterized by global reduced cerebello-cortical temporal variability with the notable exception of crus 2 whose FC with SN was enhanced. Crus 2 could be considered as a hub dually and differentially influencing intra-network nodes. The elucidation of these effects are particularly relevant given the major implication of the neocerebellum in cognitive operations [36]. Our results reinforce the notion that cerebellar circuitry is a major site for internal models, According to this leading theory, expectations and estimates of future motor or cognitive states are critical for performing motor or mental operations [37]. These internal models require updates on a constant basis. Temporal variability can be seen as one of the parameters tuned by the neocerebellum.

## Data Availability

data and materials could be provided by authors on request.
